# Growth of Tropical dasyatid Rays Estimated Using a Multi-Analytical Approach

**DOI:** 10.1371/journal.pone.0077194

**Published:** 2013-10-11

**Authors:** Owen R. O’Shea, Matias Braccini, Rory McAuley, Conrad W. Speed, Mark G. Meekan

**Affiliations:** 1 Australian Institute of Marine Science, Crawley, Western Australia, Commonwealth of Australia; 2 School of Veterinary and Life sciences, Murdoch University, Perth, Western Australia, Commonwealth of Australia; 3 Shark and Ray Sustainability Group, WA Fisheries and Marine Research Laboratories, Hillary’s Boat Harbour, Western Australia, Commonwealth of Australia; University of Canterbury, New Zealand

## Abstract

We studied the age and growth of four sympatric stingrays: reticulate whipray, *Himanutra uarnak* (n=19); blue mask, *Neotrygon kuhlii* (n=34); cowtail, *Pastinachus atrus* (n=32) and blue-spotted fantail, *Taeniura lymma* (n=40) rays at Ningaloo Reef, a fringing coral reef on the north-western coast of western Australia. Age estimates derived from band counts within sectioned vertebrae ranged between 1 and 27 years (*H. uarnak*, 1 - 25 yrs.; *N. kuhlii*, 1.5 - 13 yrs.; *P. atrus*, 1 - 27 yrs. and *T. lymma*, 1 -11 yrs.). Due to limitations of sample sizes, we combined several analytical methods for estimating growth parameters. First, we used nonlinear least squares (NLS) to identify the growth model that best fitted the data. We then used this model, prior information and the data within a Bayesian framework to approximate the posterior distribution of the growth parameters. For all species the two-parameter von Bertalanffy growth model provided the best fit to size-at-age datasets. Based on this model, the Bayesian approach allowed the estimation of median values of *W*_D∞_ (cm) and *k* (yr^-1^) for the four species (*H. uarnak*: 149 and 0.12; *N. kuhlii*: 42 and 0.38; *P. atrus* 156 and 0.16, and *T. lymma* 33 and 0.24, respectively). Our approach highlights the value of combining different analytical methods and prior knowledge for estimating growth parameters when data quality and quantity are limited.

## Introduction

Elasmobranchs face increasing fishing pressure on a global scale due to a combination of rising consumer demand and life history characteristics that make them vulnerable to overfishing [1,2]. In Australian waters, batoids have been largely overlooked by researchers and managers involved in commercial fisheries primarily due to their low commercial value in comparison to sharks. However, they are still significant components of fisheries bycatch, particularly in penaeid fisheries [3]. In a wider Indo-Pacific context, many demersal rays such as dasyatids are targeted in artisanal and small-scale fisheries for their meat and leather [4] yet for the most part, there is a lack of even the most basic life-history information for these taxa. Given that knowledge of growth rates and age structures are essential for determining the ability of populations to sustain and recover from overfishing, studies on the age and growth of batoids are urgently required. 

Chondrichthyans have been aged by counting growth band pairs in vertebrae for over 90 years [5]. While such techniques are generally reliable, accurate and common-place, the acquisition of adequate sample sizes remains a major challenge [6], particularly for those species that are poorly represented in commercial fisheries (the most common method for sourcing specimens) [7] due to gear selectivity [8,9] and/or spatially/temporally restricted sampling [10]. Low sample sizes hinder age and growth studies because they present challenges for the estimation of growth parameters using conventional analytical approaches. For example, low sample sizes may result in techniques such as nonlinear regression providing estimates that are not an accurate reflection of growth patterns [11]. Combining analytical methods to increase accurate estimation in such cases is useful and using a Bayesian framework is one such approach which can aid in overcoming these issues by guiding the estimation of parameters through the use of prior knowledge [11]. 

Here, we determined the ages and growth parameters of four abundant stingrays in a coastal, coral reef environment at Ningaloo Reef, Western Australia. Our approach combined different analytical methods for estimating model parameters in a situation where only limited sample sizes were available. We provide the first account of age and growth parameter estimates for these rays at Ningaloo Reef, including three species for which no age and growth information has previously been reported. Our approach has relevance to studies of other elasmobranchs where collection sizes may be restricted due to the difficulty of sampling (e.g. deep sea and no-take areas) or the rarity and/or protected status of the subject animals. 

## Materials and Methods

### Study sites and sample collection

**Figure 1:**
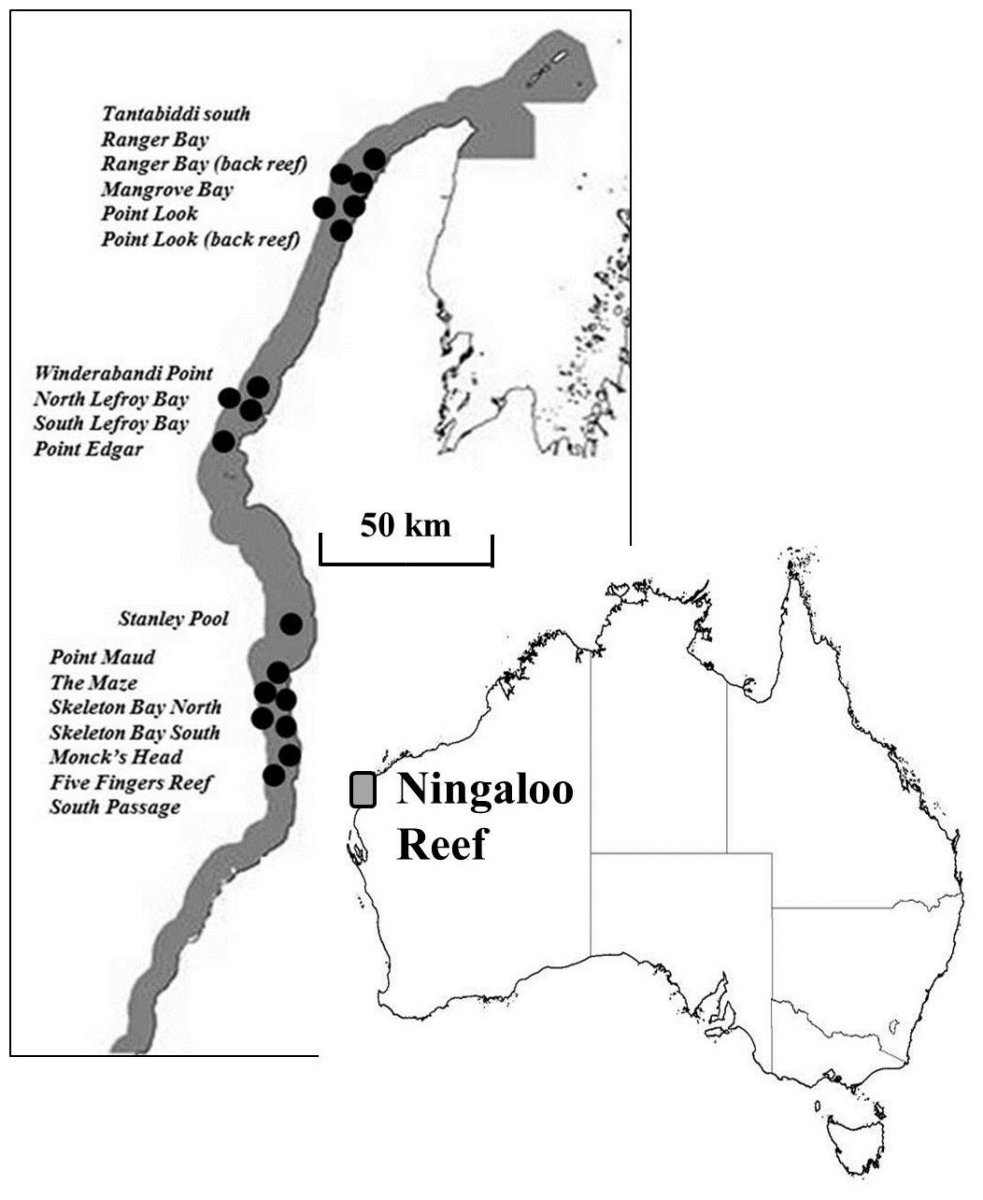
Map of the eighteen sites accessed for sampling within the Ningaloo Reef Marine Park, Western Australia.

A total of 170 individuals (*H.uarnak*, Forsskål 1775, n=24; *N. kuhlii*, Müller and Henle, 1841, n=36; *P. atrus*, Macleay 1883, n=43; *T. lymma*, Forsskål 1775, n=54 and *U. asperrimus*, Bloch and Schneider 1801, n=13) were collected for aging between February 2010 and February 2011 in the shallow (2‒10 m water depth) lagoons of the Ningaloo Reef Marine Park (Figure 1). Due to logistical constraints, sampling was restricted to the months of February (38% of total catch) and August and September (62%). Small rays were caught with hand nets and larger individuals were caught using spear guns following methods outlined in [12]. Logistic, environmental and ethical constraints resulted in small sample sizes, in contrast to other studies that have been able to use large seine nets over sand flats or sourced individuals from commercial fishers [13]. This was not possible at Ningaloo Reef where commercial fishing activities are not permitted within the marine park and the lagoon and nearshore intertidal areas are dominated by coral reef. All animals were collected under WA fisheries exemption permit # RS457/98-05 and Murdoch University animal ethics licence #U6/2010-2011 and Murdoch University ethics permit #R2275/09.

For age validation, fifty-two individuals (*H. uarnak*, n=8; *N. kuhlii*, n=11; *P. atrus*, n=19; and *T. lymma*, n=14) were caught at Skeleton (n = 30; 23° 8.378’S 113° 46.240’E) and Mangrove (n = 22; 21°58.385’ S 113°56.99’ E) Bays in the Ningaloo Marine Park between November 2009 and November 2010. Each ray was weighed, measured (disc width, *W*_D_, and total length, *T*_L_), fitted with a T-bar spaghetti tag, injected with calcein at 3-ml/kg body weight and then released. 

### Vertebrae preparation

Vertebrae were removed posterior to the cranium at the widest point of the animal and stored in a freezer within 8 hours of excision for transport to the laboratory. In the lab, centra were cleaned of connective tissue before being placed in a 5% sodium hypochlorite solution for between 0.5 - 2 hours depending on their size. The samples were then soaked in distilled water for ten minutes before being air-dried overnight. Next, three centra were embedded in clear polyester casting resin and left to set overnight, after which sagittal sections (350 µm) were cut from the resin blocks using an isomet 2000 linear precision saw. Sections were placed under a dissecting microscope and covered in methyl salicylate liniment APF to remove imperfections and cracks created by the saw. Each centrum was photographed under reflected light up to five times using a mounted camera. Images were edited using QuickTime (V.7.6.6) image capture software. 

### Age Estimation

**Figure 2:**
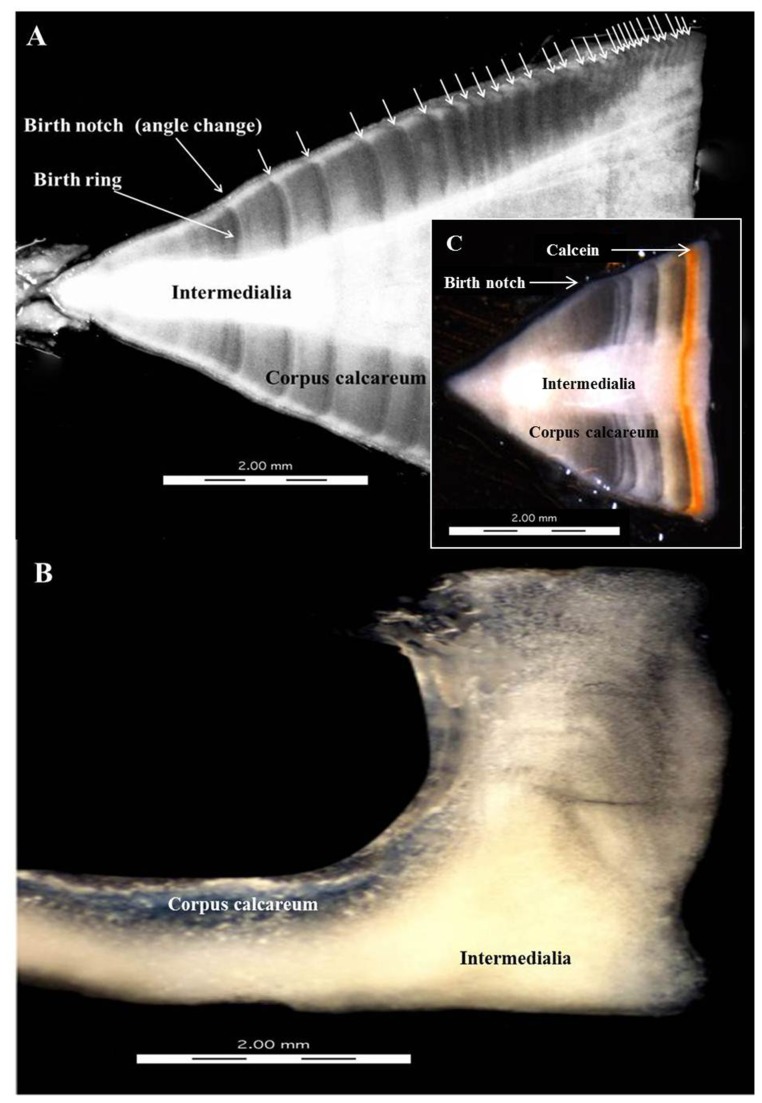
Photographic images of sagittal centrum sections from (A) *Pastinachus atrus* with 27 band pairs (B), example of difficult to read section from *Urogymnus asperrimus* and (C) example of calcein marked centra from 91 days at liberty for *P. atrus*.

Alternating opaque and translucent bands representing one band pair were visible in all samples with the exception of those from *U. asperrimus*. For this latter species, no further analysis was possible. The position of the birthmark in the section was evaluated from the angle change on the outer edge of the corpus calcareum [14] (Figure 2A). Pre-birth banding was not present in any of the neonate samples; consequently the first band pair was regarded as age one. Age was determined by counting the band pairs on the outer edge of the corpus calcareum and 0.5 years was added if a translucent or opaque band was forming on the outer centrum edge [15,16]. Biases in sampling effort between seasons and unconventional band formation within seasons (i.e. both translucent and opaque bands formed in each season) justified this approach. Two training counts were conducted to achieve fluency in interpreting banding pairs but these scores were not included in the final results. Three blind, independent counts were then made of each sample using three different readers. Final age estimates were achieved when the same age estimate was obtained from two or more readers. A qualitative readability score from one to three was given to each sample, where one meant all bands were clear and unambiguous; two, bands visible but difficult to interpret; and three, bands were unreadable (modified from [17]). Samples (n=45) with readability scores of 3 were excluded from the analyses. The index of average percentage error (IAPE) (Table 1) was calculated, after Beamish & Fournier [18], to estimate the precision of age determination among readers. When averaged across multiple counts for multiple rays, the index provided an estimate of average percent error [19]. In addition, the coefficient of variation (CV) [20] was also calculated (Table 1). 

**Table 1:** Index of average percentage error (IAPE) and coefficient of variance (CV) values for inter-reader precision of age determination (*i* = reader).

**Species**	**IAPE *i*=1**	**IAPE *i*=2**	**IAPE *i*=3**	**CV *i*=1**	**CV *i*=2**	**CV *i*=3**
Himantura uarnak*	1.69	1.66	2.92	2.64	2.29	4.03
Neotrygon kuhlii**	2.19	2.84	5.25	3.12	4.04	7.47
Pastinachus atrus*	1.43	2.45	4.66	2.07	3.55	6.75
Taeniura lymma**	2.09	2.15	3.61	2.80	2.88	4.84

* denotes larger bodies species, ** *smaller bodies species*

### Growth parameter estimation

We used a three-step approach to optimise the estimation of growth parameters of each species. First, we pooled male and female samples and used Ford-Walford plots [21,22] to determine adequate starting values for parameter estimation. We then used nonlinear least squares to compare a range of growth models to determine if a particular model best described the growth data [23]. Estimated ages and observed sizes (*W*_D_), were fitted to four commonly used models (Table 2): the three-parameter von Bertalanffy (VBGF) [24,25], the modified two-parameter von Bertalanffy (2VBGF) [26],, the logistic (after [9]) and the three-parameter Gompertz (GGF) [27]. Akaike’s information criterion (AIC) with a bias correction (AIC_*c*_) due to small sample sizes (<200) was used to determine the best model fit [9,28]. Models were ranked according to AIC differences (Δ) where models with a Δ value of between zero – two were considered to have the highest support, while any higher Δ values were considered to have lower support [29]. 

**Table 2:** Growth models and associated formulas used to fit size-at-age data for four species of dasyatid rays.

**Model**	**Growth Function**
3 parameter von Bertalanffy (VBGF)	*W*_D_ *t* = *W*_D∞_ [1 - *e* ^–k (t-t0)^]
2 parameter von Bertalanffy (2VBGF)	*W*_D_ *t* = *W*_D∞_ (1 - *be * ^*–kt*^), *b* (*W*_D∞_ *W*_D0_)/*W*_D∞_
Logistic (LOG)	*W*_D_ *t* = ((*W*_D∞_ *W*_D0_ *e*) */* (*W*_D∞_ + *W*_D0_ (*e* ^(kt)-1)^))
Gompertz (GGF)	*W*_D_ *t* = *W*_D∞ =_ *e* ((-*W*_D0_ *e* (-*kt*))

Once the model with the best fit was determined, we adopted a Bayesian approach with a penalised likelihood to approximate the posterior distribution of the growth parameters for each of the species (see justification for adopting a Bayesian approach in Appendix S1). Markov Chain Monte Carlo (MCMC) methods using the Metropolis Hastings algorithm were used to sample the posterior distributions [11,30,31]. We used a chain of two million iterations with a burn-in period of 100,000. Owing to the high auto-correlation in the MCMC chain, we used a thinning of 100. We used informative priors for *W*_D∞_ (asymptotic size expressed as disc-width) and *K* (yr^-1^) (growth coefficient – the rate at which asymptotic size was reached) based on all recently published estimates for sub-tropical/tropical dasyatid species (n=7) that were also derived from vertebral sections [13,15,32-35]. Given that age and growth in dasyatids may not conform to the general pattern that larger species live longer and grow more slowly when compared to smaller species [13], we decided to use one prior for each parameter that took large and small species into consideration as opposed to setting one prior for large and one for small species. The prior for *W*_D∞_ was lognormal with mean 77 cm and standard deviation of 0.5 (in log space). We used a beta distribution as a prior for *K* (yr^-1^) (Beta; 21.9; 162.3). The prior on the variance term was non-informative, defined by an inverse Gamma distribution (IGamma 0.01, 0.01). Preliminary sensitivity tests using informative or non-informative priors for *W*_D∞_ and *K* (yr^-1^) showed that the data were able to update the priors. Evidence of convergence of the MCMC chains was warranted by standard convergence diagnostics (visual inspection of the trace plots, the Geweke diagnostic test and from comparing summary statistics for the first 10% of the chain and the second half of the chain). All analyses were conducted using the statistical package R [37]. 

## Results

Of the 170 rays sampled, the vertebrae of 29% (n=50) achieved readability scores of one, while 44% (n=75) achieved scores of two, and 26% (n=45) were assigned scores of three. The 13 *U. asperrimus* vertebral samples were excluded from analyses with only one sample attaining a readability score of <3. In this species, the cartilaginous matrix of the centra was very brittle and it was therefore problematic to obtain accurate counts of band pairs (Figure 2B). The remaining samples that proved difficult to age were typically from very small individuals and full term pups, where calcification within the centra was either insufficient to obtain counts or was not present. The index of average percentage error (IAPE) and coefficient of variation (CV) for the selected samples generally showed low inter-reader variability, particularly for the larger bodied species (*H. uarnak* and *P.atrus*) compared to the two smaller species (*N.kuhlii* and *T. lymma*) (Table 1). 

### Recaptures and seasonal edge deposition

Of the 52 rays caught and marked with calcein, only two *P. atrus* individuals were recaptured after 83 and 91 days. These rays had both grown 5 cm (*W*_D_) during this period and both had laid down translucent bands of 0.2 cm in width after a very pronounced calcein mark (Figure 2C). Unfortunately, the time at liberty was insufficient for validation of band pair periodicity. Variation in sampling effort resulted in 48 rays being caught in summer and 77 in winter. Of the 48 individuals caught in February, 28 (58%) had opaque bands forming at the edge of the centra, while during the winter months, 51 rays (66%) had translucent bands forming on the centrum edge. 

### Estimation of ages

**Figure 3:**
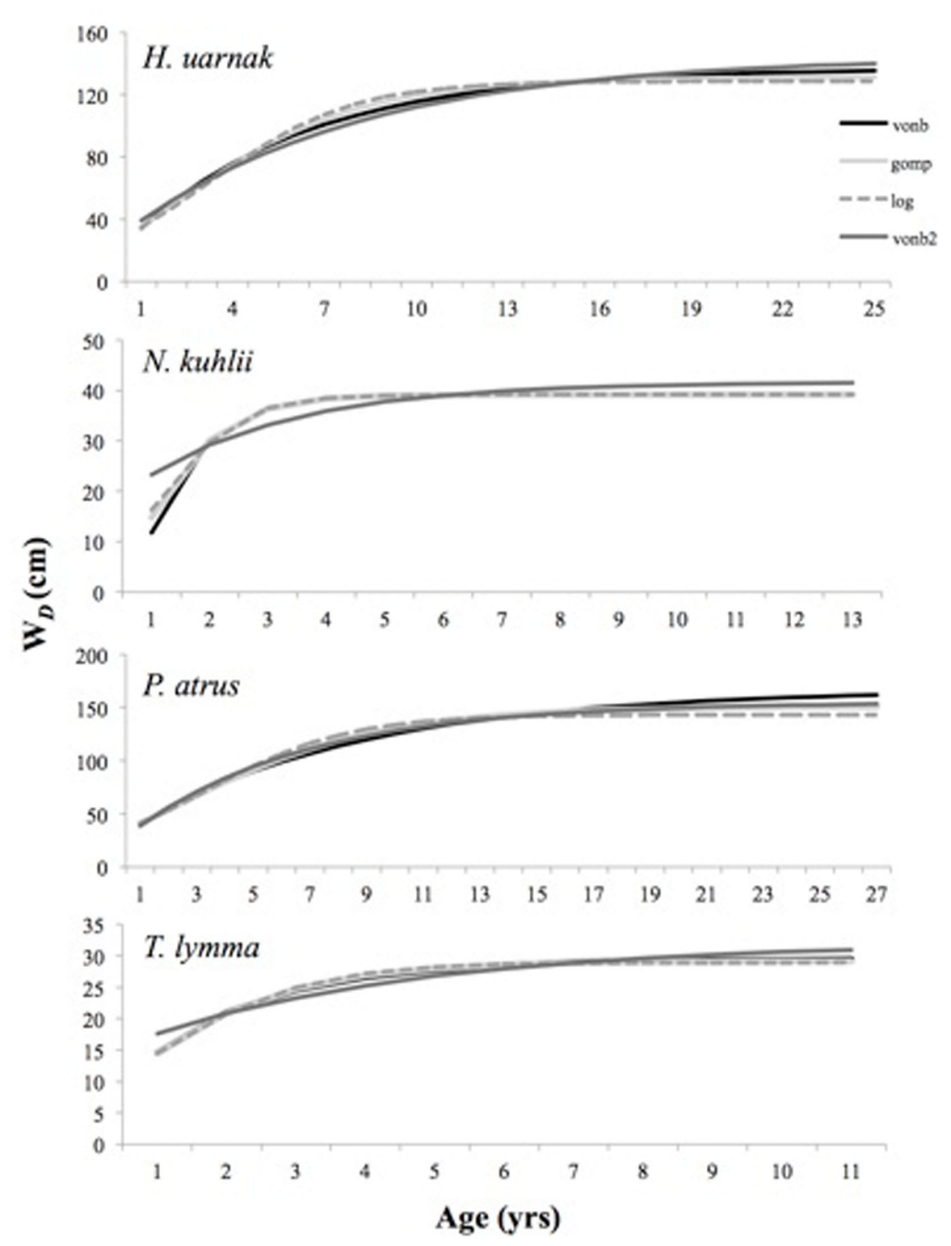
Predicted size at age from four growth models: Vonbertalanffy, Gompertz, Logistic and two-parameter Vonbertalanffy.

Estimated ages ranged from one to 25 years for *H. uarnak* (25–145.4 cm *W*_D_), 1.5 to 13 years in *N. kuhlii* (17–47 cm *W*_D_), one to 27 years in *P. atrus* (36.5–177 cm *W*_D_), and one to 11 years in *T. lymma* (14–34.5 cm *W*_D_) (Figure 3). According to AIC, the 2VBGF was the model of choice, though the other models show very similar fits (Table 3 and Figure 3). 

**Table 3:** Comparison of growth model’s fit and parameter estimates.

**Species**	**Model**	**ΔAIC**	***W*_D∞_ (cm**)	***K* (yr ^-^¹**)	***T*_0_ (yr**)
***Himantura uarnak***	VBGF	3.03	137	0.17	0.68
**N=19**	GGF	3.93	131	0.29	1.99
	LOG	4.34	128	25	0.43
	2VBGF	0	145	0.13	NA
***Neotrygon kuhlii***	VBGF	3.03	39	1.12	0.68
**N=34**	GGF	2.86	39	1.29	0.98
	LOG	2.87	39	5.45	1.48
	2VBGF	0	42	0.38	NA
***Pastinachus atrus***	VBGF	2.49	167	0.12	-1.25
**N=32**	GGF	2.32	151	0.24	2.12
	LOG	2.87	74	30.35	0.39
	2VBGF	0	155	0.16	NA
***Taeniura lymma***	VBGF	2.77	29	0.58	-0.22
**N=40**	GGF	2.81	29	0.73	0.5
	LOG	2.83	29	8.29	0.92
	2VBGF	0	32	0.25	NA

### Estimation of growth parameters

**Figure 4:**
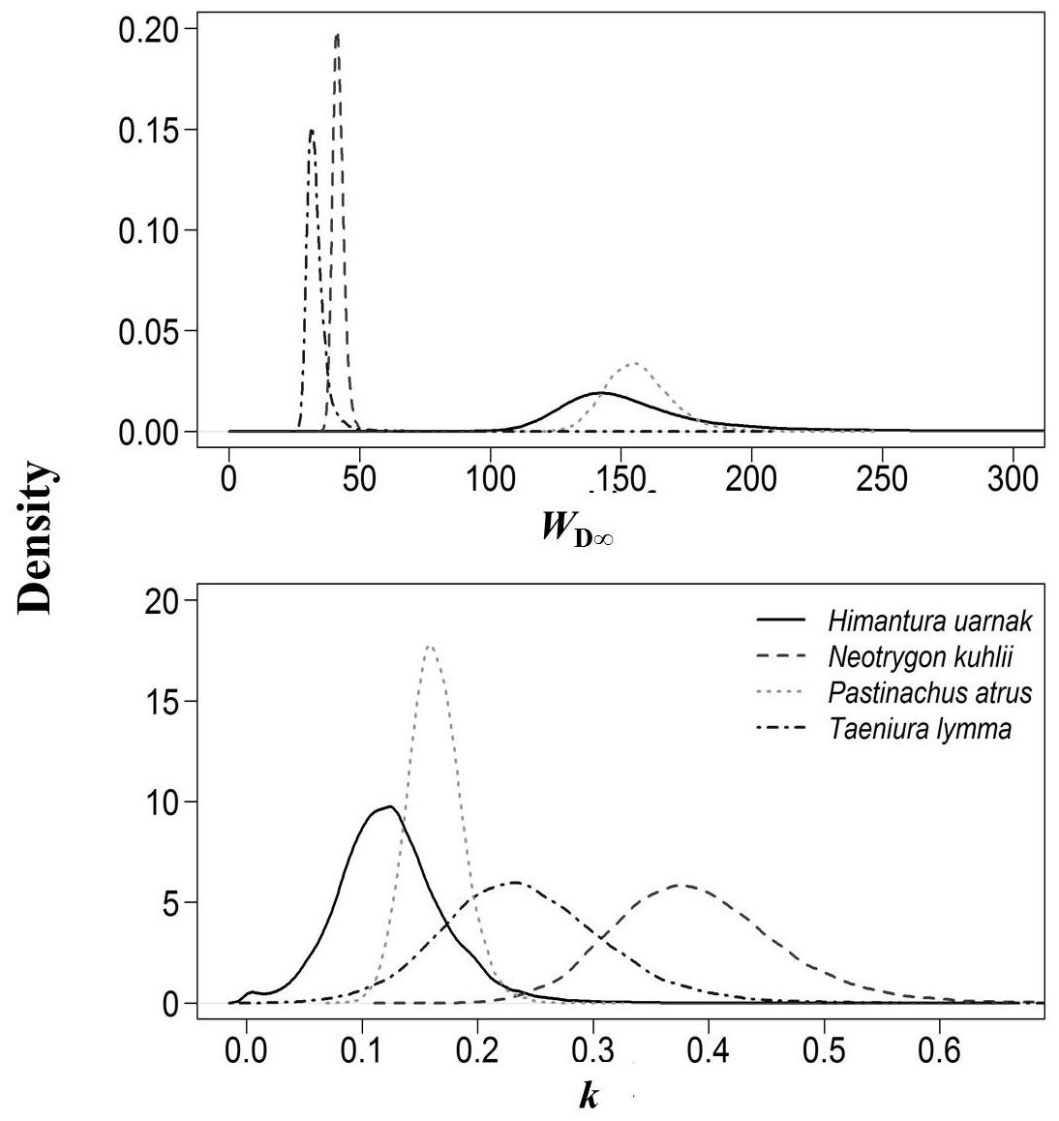
Posterior distributions for *W*_D∞_ and *k*, for the four species where band pairs could be counted.

Diagnostic tests indicated MCMC chain convergence for all growth parameters for three species (*N. kuhlii, P. atrus and T. lymma*). Convergence for *W*_D∞_ for *H. uarnak* was less obvious, reflecting the less than ideal nature of the data (i.e. few large/old individuals). Growth data were informative for all species, updating the priors for *K* (yr^-1^) and *W*_D∞_ in all cases (Figure 4). The Bayesian approach provided more precise estimates of *K* (yr^-1^ median with 95% credibility intervals) for *P. atrus* (*K* = 0.16 yr^-1^, 0.12‒0.21 yr^-1^) than for the remaining species (*H. uarnak: K* = 0.12 yr^-1^, 0.04‒0.22 yr^-1^; *N. kuhlii*: *K* = 0.38 yr^-1^, 0.25‒0.53 yr^-1^; and *T. lymma*: *K* = 0.24 yr^-1^, 0.1‒0.38 yr^-1^). For *W*_D∞_, more precise estimates were obtained for *N. kuhlii*, (*W*_D∞_=42 cm, 38–46 cm) and *T. lymma*, (*W*_D∞_=33 cm, 28–41 cm) than for *P. atrus* (*W*_D∞_=156 cm, 133–181 cm) and *H. uarnak* (*W*_D∞_=149 cm, 107–231 cm), with the latter showing a much broader posterior distribution of values (Table 4 and Figure 4). 

**Table 4:** Summary of growth parameter estimates (median with 95% credibility intervals) from the Bayesian modelling approach.

**Species**	***W*_D∞_ (cm) ± 95% C.I.**	***K* (yr ^-^¹) ± 95% C.I.**
***Himantura uarnak***	149 (107 - 231)	0.12 (0.04 - 0.22)
***Neotrygon kuhlii***	42 (38 - 46)	0.38 (0.25 - 0.53)
***Pastinachus atrus***	156 (133 - 181)	0.16 (0.12 - 0.21)
***Taeniura lymma***	33 (28 - 41)	0.24 (0.1 - 0.38)

## Discussion

Our study shows that growth parameters of tropical dasyatids can be estimated in data-limited situations using a combination of statistical methods, a result relevant for other studies of elasmobranchs particularly where the species of interest are rare and/or protected, or occur in areas where conventional methods that yield large sample sizes may not be used. Importantly, our samples included a range of size classes, enabling the estimation of growth parameters without the need to resort to other methodologies (e.g. back calculation). 

### Validation of age estimates

Although we attempted age validation through the recapture of chemically-marked individuals [19] we failed to obtain any rays after a sufficient period at liberty. For this reason we assumed that band pairs were deposited on an annual basis in order to analyse growth patterns. This approach appeared reasonable given that annual patterns of deposition within vertebrae have been reported for the majority of elasmobranchs examined to date [14]. 

### Growth models and parameter estimates

While the use of an information criterion method (AIC) suggested that the two-parameter von Bertalanffy function (2VBGF) provided the best fit to growth data of all species, selection was only marginal and other growth models we trialled showed similar fits. Parameter estimates from the 2VBGF model were also comparable to recently published estimates for other tropical dasyatid rays aged in the same manner [13,15,32-37]. Given that *L*_0_ is generally well documented for sharks and rays, the use of the 2VBGF – where only the *k* and *W*_D∞_ parameters were estimated – was an intuitive choice over the traditional VBGF model, particularly since sample sizes were small and some age classes, particularly younger individuals, were poorly represented [6,8]. Due to the requirement of estimating an additional parameter, the highly correlated nature of growth parameters and the less than ideal representation of age and size data, it was not surprising that the 2VBGF outperformed the other models when ranked using AIC. However, since parameter estimates derived from this model may under-estimate *W*_D∞_ and overestimate *k* (yr^-1^), our results should be treated with caution [8]. 

Underlying biases within age and growth data are a common problem for studies of elasmobranchs due to factors that affect the sample collection process such as selectivity of sampling gear and the heterogeneous spatial and temporal patterns of abundance of this mobile group of animals. In our study, even though sample sizes were small and not all age classes were well represented, the application of a multi-staged method based on the 2VBGF and Bayesian estimation allowed a reasonable approximation of the growth parameter metrics. These estimates were close to those obtained through a nonlinear least squares (NLS) analysis however, the NLS was more sensitive to the initial parameter values used in the estimation and it produced more uncertain estimates of growth parameters (Appendix A).

Studies of related species suggest that no particular growth model outperforms any other when describing the growth of dasyatids. For brown stingrays (*Dasyatis lata*) the LOG growth model provided the best fit to age at size data [37], whereas growth of the black whipray (*Himantura astra*) [13] and the diamond stingray (*Dasyatis dipterura*)[15] were described with greater certainty by GGF and the VBGF models respectively. Given that outputs from age and growth studies are limited by their sample size, size range distribution, validation techniques and model constraints [6,15,38], a preferred growth model and parameter estimates can exhibit considerable variation among both studies and species of the same family, as is the case for urolophids [41-44] 

Growth rates are defined by the growth coefficient (*k* yr^-1^) that describes the rate at which growth slows as the animal ages [1]. Slow-growing elasmobranchs are defined as having *k* (yr^-1^) values <0.1 [38] and it is assumed that these species are more vulnerable to extrinsic pressures such as overfishing [39] than those faster-growing species where *k* (yr^-1^) >0.1. Thus, our findings suggest that of the two largest species we studied, *H. uarnak* was more vulnerable as a slower-growing species (*k* yr^-1^ = 0.12), than *P. atrus* (*k* yr^-1^ = 0.16), though their posterior distributions overlapped considerably. As expected, the two smaller-bodied species (*N. kuhlii* and *T. lymma*) both had faster growth rates (*k* yr^-1^ =0.38 and 0.24 respectively) and thus may be less vulnerable. Published studies for other sub-tropical/tropical dasyatid species show similar results, with those species attaining larger maximum sizes (*W*_D_max >100cm) having slower growth rates than smaller-bodied species (e.g. [13,40]). 

## Conclusions

The development of methods that produce accurate and robust estimates of growth parameters of elasmobranchs when sample sizes are small and may not be representative of the entire population is critical for determining the conservation status of rare and protected taxa, or any species where the collection of large sample sizes presents logistic or ethical problems. By combining different analytical methods and maximising the use of available information, our approach increased the precision of estimates of growth parameters of tropical shallow water rays. 

## Supporting Information

Figure S1 **Simulated data sets.** The upper panel shows the well-represented data set (10 observations for each of the 20 age classes) from where samples were drawn. The middle and lower panels show an example of data-poor sampling.
(TIF)

Figure S2 **Comparison of the performance of nonlinear least squares (NLS) and Bayesian methods for estimating growth parameters based on simulated data.** The broken line indicates the parameter value used for simulating the data.
(TIF)

Appendix S1 **Performance of NLS and Bayesian methods when estimating growth parameters in data-poor cases.**
(DOCX)
