# Supplementary figures and images for: Growth of Tropical dasyatid Rays Estimated Using a Multi-Analytical Approach
Source: PLoS One. 2013 Oct 11;8(10):e77194. doi: 10.1371/journal.pone.0077194 (PMC3795619; doi:10.1371/journal.pone.0077194)

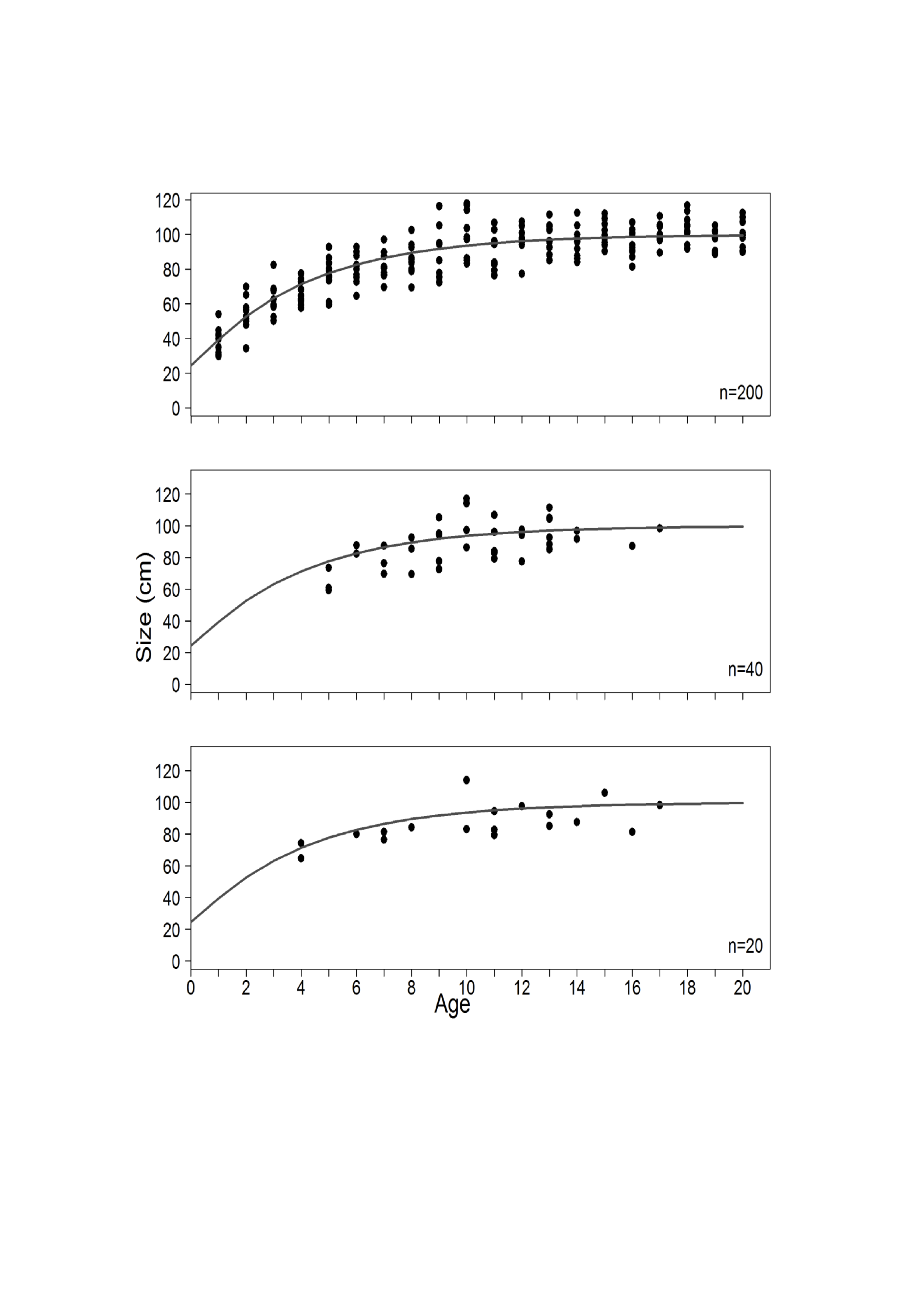

Supplement: Figure S1 — Simulated data sets. The upper panel shows the well-represented data set (10 observations for each of the 20 age classes) from where samples were drawn. The middle and lower panels show an example of data-poor sampling. (TIF) [file pone.0077194.s001.tif]

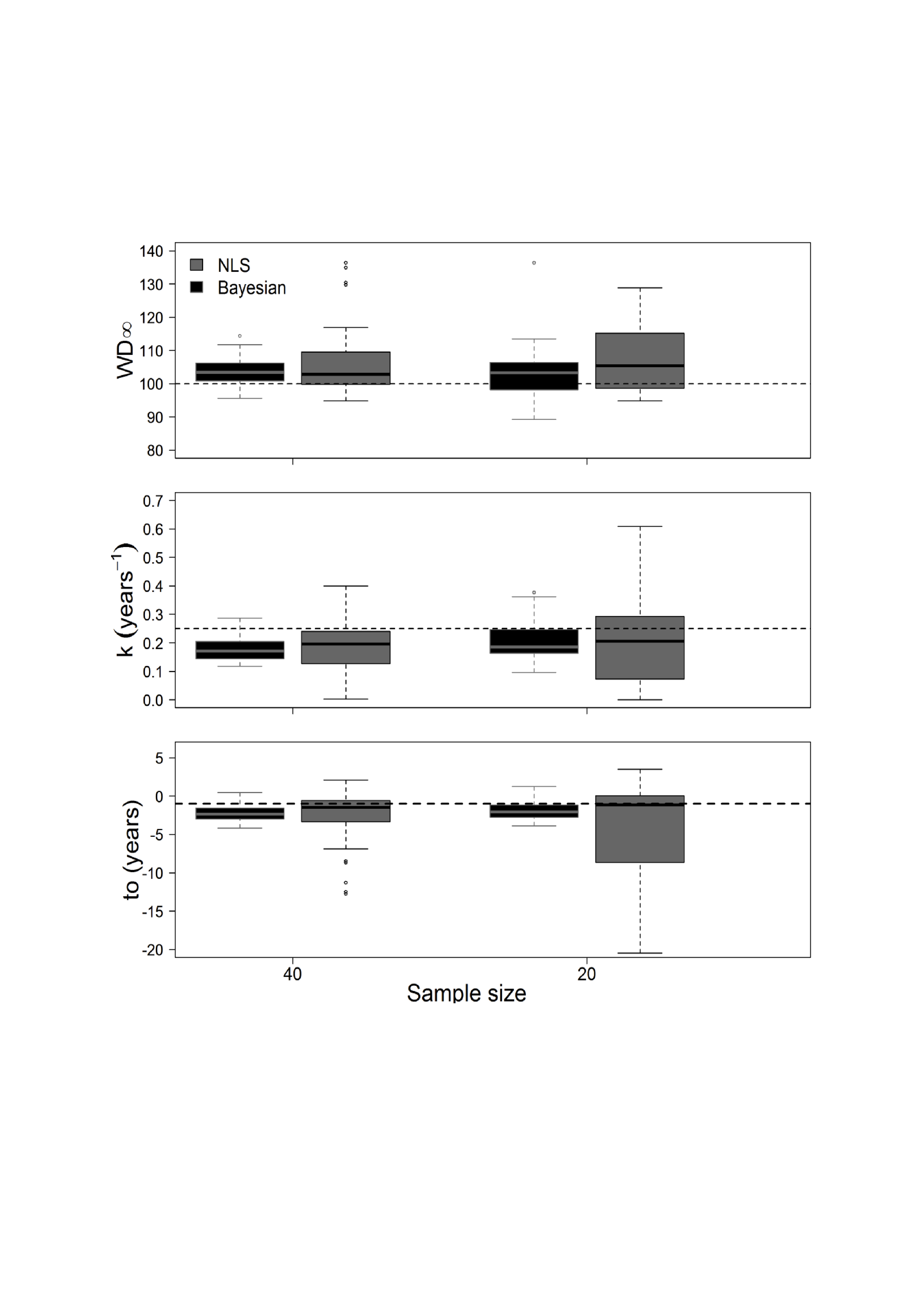

Supplement: Figure S2 — Comparison of the performance of nonlinear least squares (NLS) and Bayesian methods for estimating growth parameters based on simulated data. The broken line indicates the parameter value used for simulating the data. (TIF) [file pone.0077194.s002.tif]
