# Supplementary material for: Growth of Tropical dasyatid Rays Estimated Using a Multi-Analytical Approach
Source: PLoS One. 2013 Oct 11;8(10):e77194. doi: 10.1371/journal.pone.0077194 (PMC3795619; doi:10.1371/journal.pone.0077194)
Supplement: Appendix S1 — Performance of NLS and Bayesian methods when estimating growth parameters in data-poor cases. (DOCX) [file pone.0077194.s003.docx]

**Appendix S1.**

Ideally, justification of the use of a particular parameter-estimation method should be based on the comparison of parameter estimates obtained by different methods with those generated by independent studies. Unfortunately, such estimates were not available and for this reason we chose a simulation approach to compare the performance of Bayesian and nonlinear least squares (NLS) techniques. The latter is a method used commonly for estimating growth parameters of sharks, rays and teleosts. First, we simulated a representative sample of size-at-age data using a traditional three-parameter von Bertalanffy growth model, 20 age classes and the parameter values of WD∞ =100 cm, *K*=0.25 yr -¹ and *T*_0_= -1 yr. For each of the age classes, ten size observations were generated using a normal distribution with mean given by the size-at-age calculated from the von Bertalanffy growth model and standard deviation of 10 cm. This generated a data set of 200 observations (Figure S1). Next, two data sets of 40 and 20 observations were created by sampling the 200 observations of the representative sample case. We simulated biased, size-selective sampling by taking random samples with probability-at-age given by a dome-shaped curve with 100% selectivity at age=10 years and decreasing selectivity for younger and older age classes. This is a common situation for many age and growth studies of shark and rays where age classes are not equally represented due to the use of selective sampling gear, spatial/temporal heterogeneity in size distribution, and/or opportunistic sampling. A total of 50 data sets were generated for the 40- and 20-observation cases (Figure S1). Growth parameters were then estimated by fitting the three-parameter von Bertalanffy growth model to these data sets using NLS and Bayesian methods. Figure S2 shows that overall the Bayesian approach shows a better performance than the NLS method, particularly for the 20-observation case. Though the median values obtained from the Bayesian approach were not substantially closer to the true value than the NLS medians, for the NLS and 20-observation case, model convergence was very sensitive to the initial parameter values, whereas the Bayesian approach showed more stable performance. Therefore, this approach was chosen over the traditional NLS for the estimation of growth parameters of the four dasyatid rays.
